# An Unusual Postoperative Complication Following Bilateral Inguinal Hernioplasty: A Pocket Hernia Case Report

**DOI:** 10.7759/cureus.61589

**Published:** 2024-06-03

**Authors:** Luis Carlos Lozano-Carrillo, Humberto Meléndez-Mondragón, Luis Adrian Alvarez-Lozada, Alejandro Quiroga-Garza, Juan Manuel Valdivia-Balderas

**Affiliations:** 1 Department of Human Anatomy, Clinical-Surgical Research Group (GICQx) Universidad Autonoma de Nuevo Leon, School of Medicine, Monterrey, MEX; 2 Department of Human Anatomy, Anatomy Research Group (GIA) Universidad Autonoma de Nuevo Leon, School of Medicine, Monterrey, MEX; 3 General Surgery Division, Mexican Social Security Institute, Nuevo Leon Delegation, Monterrey, MEX

**Keywords:** peritoneal hernia, pocket hernia, postop complication, postoperative small bowel obstruction, transabdominal preperitoneal (tapp), inguinal hernia, case report

## Abstract

Inguinal hernias are the most common type of hernias in the groin, affecting 27% of the population, with a nine to 12 times higher incidence in men. The primary treatment for this condition typically involves a surgical procedure, with most surgeons opting for mesh placement through a laparoscopic approach. While this procedure is generally associated with low complication rates (primarily hematomas, seromas, and scrotal edema), there are some highly infrequent complications reported such as postoperative small bowel obstruction (SBO), estimated to occur in approximately 0.1%-0.5% of cases, most commonly during transabdominal preperitoneal (TAPP) repair. It is crucial to emphasize the importance of using skilled surgical techniques and adhering to established guidelines in postoperative patient care to minimize the risk of these complications. We describe a case of a 47-year-old male patient who underwent bilateral TAPP repair for inguinal hernias and subsequently experienced postoperative complications, including the development of a hematoma and SBO, requiring a re-intervention that evidenced a peritoneal pocket hernia.

## Introduction

Inguinal hernias are prevalent, affecting 27%-43% of men and 3%-6% of women worldwide annually, with surgical intervention being the primary management [1,2]. Minimally invasive techniques, such as transabdominal preperitoneal (TAPP) repair and totally extraperitoneal repair, offer advantages, including reduced complications and faster recovery [3]. However, some surgeons hold the opinion that mesh use should be avoided as much as possible or prefer an open approach, especially in a low resources/income setting [1,2]. Despite advances, hernia recurrence rates remain between 1% and 11% [4-6]. Uncommon complications, such as pocket hernias and small bowel obstruction (SBO) post-TAPP, are reported (0.2%-0.5% of cases) [7-10]. This case highlights the need for thorough assessment and monitoring post-hernia surgery, adhering to Surgical CAse REport (SCARE) guidelines [11].

## Case presentation

A 47-year-old Latino man without any relevant medical history, athletic complex, with a BMI of 21.4, underwent a scheduled laparoscopic bilateral inguinal hernia repair (IHR). Mild discomfort during moderate to intense exercise was present due to a bilateral mixed inguinal (Pantaloon) hernia, the patient was otherwise asymptomatic. Preoperative ultrasound reported inguinal hernias with direct and indirect inguinal sac protrusion into the inguinal canal, bilaterally. Preoperative laboratories were in normal ranges.

Under general anesthesia and Trendelenburg position, standard laparoscopic TAPP bilateral IHR trocar placements were used with an 11mm trocar in the umbilicus for a 30° 5mm telescope using the Hasson technique for pneumoperitoneum. Under direct visualization, two 5 mm trocars were placed bilaterally at the same level of the umbilicus at the lateral margin of the rectus abdominis. The left inguinal peritoneal flap was dissected, reducing direct and indirect hernia sacs. Non-absorbable lightweight polypropylene mesh was placed with adequate extension and coverage over both defects and ample coverage superior and inferior to the inguinal ligament. It was fixed to the muscle in its superior border using four absorbable tackers. Hemostasis and textile count were confirmed. Peritoneal flap closure was performed with continuous taper polypropylene 2-0 suture, ensuring adequate closure. Surgeons continued with the right IHR, with the same technique. Hemostasis and textile count were confirmed again, bilaterally. Aponeurosis was closed with polyglactin-910 0 suture at the umbilicus, and poliglecaprone-25 3-0 suture for all three subcutaneous and skin incisions. The procedure and postoperative recovery were uneventful. The patient was discharged the same day and managed as an outpatient.

**Figure 1:**
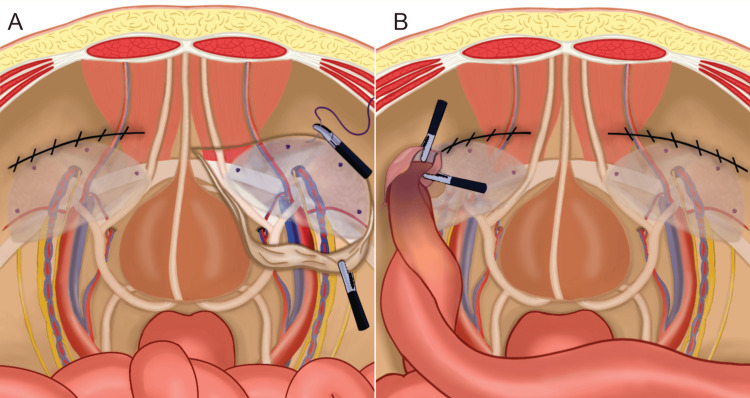
Schematic representation of laparoscopic hernioplasty. (A) First surgery. Hernioplasty with non-absorbable lightweight polypropylene mesh placement using absorbable tacker fixation, and peritoneum closure using polypropylene. Left side with peritoneal flap closed and right side open. (B) Second surgery of pocket hernia. Small bowel inside the left peritoneal flap due to opening/tear of the closure line. Courtesy: Authors

**Figure 2:**
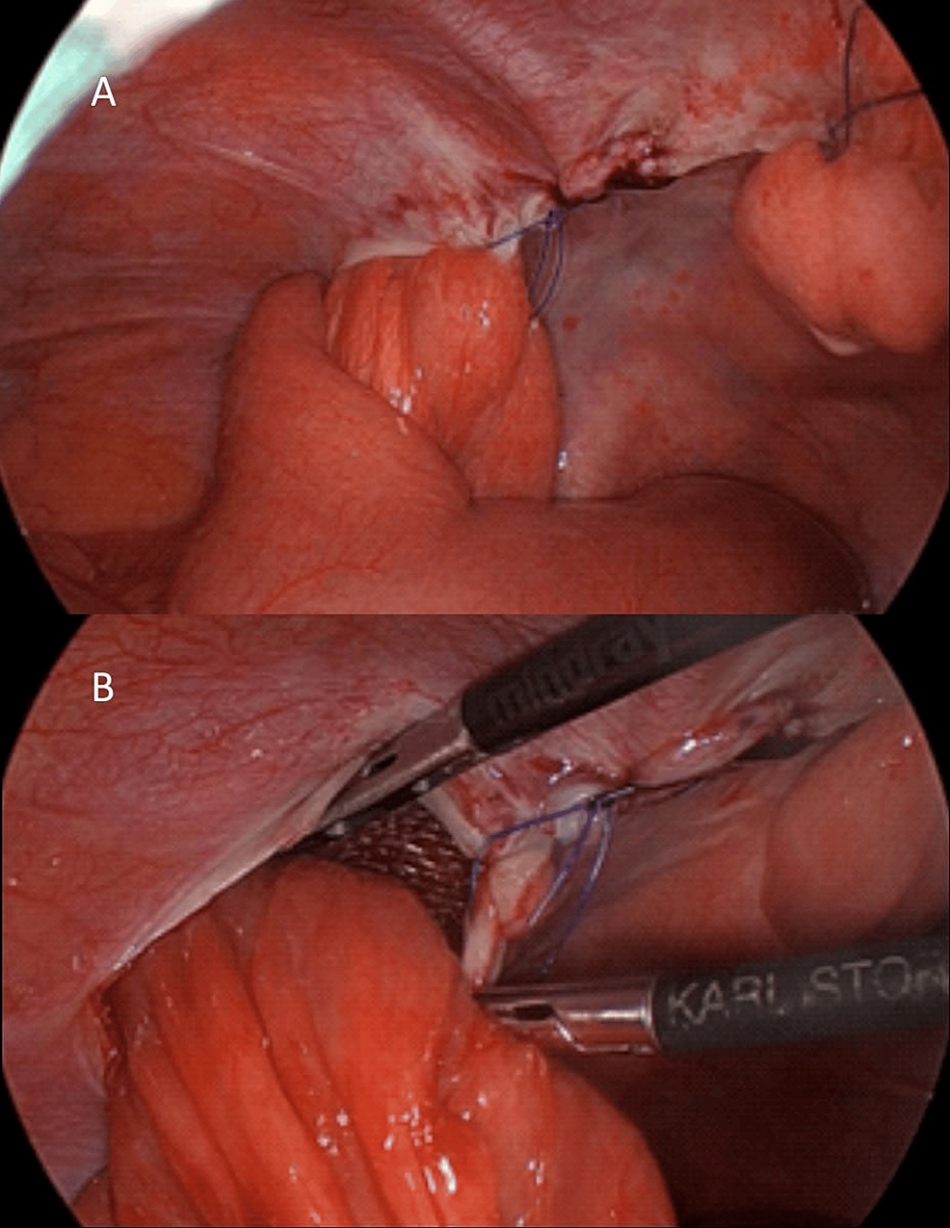
Laparoscopic view of pocket hernia. (A) Small bowel trapped inside the left peritoneal flap due to the opening in the lateral side of the closure line. (B) Peritoneal flap cut to liberate small bowel. Mesh is visible in the background, inside the flap.

On the second postoperative day, the patient arrived at the office with evidence of abnormal edema and retraction in both lateral (5mm) incisions. A small bulge in the left inguinal region was present with slight discomfort. Family members reported the patient had been active and not rested as indicated during the postoperative days. He was managed with changes in diet and medications. On the fourth postoperative day, the patient presented bloating, malaise, loss of appetite, and abdominal pain. He was referred to the emergency department. Computerized tomography revealed a small liquid collection in the left inguinal region compatible with a hematoma, and a conglomeration of small intestinal bowels next to the region with dilation of proximal bowels showing gas-fluid levels. Laboratories with WBC 13.0. Rest without important alterations. He was admitted for intravenous fluids, medical management, and a nasogastric tube. He was scheduled for emergency laparoscopic surgery using the same incisions, in which a small bowel was identified to be trapped in a peritoneal opening, lateral to the left peritoneal flap (Figures 1A, 1B). The opening was extended to liberate the small bowel, without evidence of perforation or loss of bowel wall integrity (Figures 2A, 2B). Inflammatory hemorrhagic fluid was aspirated. Mesh was visualized without evidence of migration or alteration and, therefore, left undisturbed. The peritoneal flap was closed with taper polypropylene 2-0 suture assuring complete closure. The right side was also reinforced, and a peritoneal flap tear was identified on that side. Aponeurosis and skin were closed the same way as previous surgery. The patient tolerated a liquid diet on the first postoperative day and was discharged on the third postoperative day. The seven-day follow-up visit was uneventful regarding hernias and food toleration; however, the incisions once again presented edema, retraction, and tissue hardening. The patient was referred to dermatology to evaluate the suture reaction and steroid infiltration.

## Discussion

Laparoscopy has solidified its status as the standard approach for IHR, typically resulting in good postoperative outcomes [9,12]. Nevertheless, postoperative complications have been documented [12]. A substantial discrepancy is observed in a broader examination of TAPP procedures, encompassing both unilateral and bilateral IHR. Bilateral TAPP procedures have a higher rate of complications, requiring reoperation, as revealed by multivariable analysis, underscoring the complexity of bilateral cases [3].

The incidence of SBO following a TAPP repair is approximately 0.1%-0.5% [1,13]. Only isolated cases of postoperative SBO have been documented. Potential causes encompass herniation at the trocar insertion site, intestinal adhesions, and incarceration through a prior hernia defect. In this case, we present an instance of post-TAPP intestinal obstruction accompanied by edema and retraction of surgical wounds [14]. Potential causes may be attributed to suture material or technique, which after pneumoperitoneum and ambulation may have disrupted the peritoneum. However, similar cases have been reported with tackers and interrupted sutures [15,16].

In previous cases, many had employed various techniques for mesh fixation and peritoneal closure. Non-absorbable tackers or glue have been used to secure the mesh. Peritoneal flap closure has been described with continuous or interrupted absorbable barbed sutures, non-absorbable sutures, and tackers. In our case, we used 2-0 polypropylene sutures as barbed sutures were unavailable [13,16-19]. A review of peritoneal flap closure techniques and materials is needed to determine whether these influence complication rates and other variables such as quality of life [15,16].

A potential concern with laparoscopic procedures is their ability to expose the vascular structures in the inguinal area, which includes the triangle of doom and the vascular network known as corona mortis, thereby increasing the risk of vascular injuries and potential complications. In the context of IHR, Trendelenburg positioning has been recommended to displace the bowel away from the inguinal region, improving anatomical exposure, a measure correctly employed in this case [14,20].

The postop aftercare recommendations by the European Hernia Society Guidelines state that heavy lifting and high-intensity activities must be avoided for a lapse of two to three weeks [1,12]. In this case, the patient did not follow these recommendations and returned to normal activities without precautions, which may have affected the healing process, and possible peritoneal tear causing the consequent peritoneal herniation.

Although the surgical management of this complication was performed promptly, avoiding further complications, the imaging technique influenced the decision. For patients with postoperative symptoms such as fever, nausea, vomiting, abdominal pain, and signs of peritoneal reaction, guidelines recommend abdominal x-ray and CT imaging to assess the patient [14]. In this case, the CT was conducted.

Technical recommendations to prevent SBO following laparoscopic hernia repair include ensuring the closure of all peritoneal defects and, when utilizing barbed sutures attention to excess thread, and preventing suture loosening, thus avoiding surgical technical inadequacy. Surgeons should be skillful in laparoscopic suture techniques to properly repair any peritoneal gaps that arise during surgical preparation, including those unrelated to the suture line of the peritoneal flap [16,18,21]. Furthermore, in cases of short-term post-TAPP surgery SBO, consideration should be given to possible preperitoneal herniation of the small intestine.

## Conclusions

While TAPP surgery has been documented to have rare complications, it is imperative to employ a precise surgical technique and adhere to established guidelines to minimize these risks. Additionally, ensuring high-quality postoperative patient care is crucial. In one case, postoperative bowel obstruction due to bowel herniation into a peritoneal tear was successfully managed with early laparoscopic intervention.
